# Microarray dataset supporting a role for ATF4 in isoginkgetin-induced gene expression in HCT116 cells.

**DOI:** 10.1016/j.dib.2022.108126

**Published:** 2022-04-01

**Authors:** Erin van Zyl, Victoria Tolls, Bruce C McKay

**Affiliations:** 1 Department of Biology, Carleton University; 2 Institute of Biochemistry, Carleton University

**Keywords:** ATF4, Isoginkgetin, Microarray, Spliceosome inhibition

## Abstract

Isoginkgetin (IGG) is a compound originally derived from the leaves of *Ginkgo biloba* trees. It was subsequently identified through a chemical screen to be an inhibitor of both the major and minor spliceosome, with an IC50 value of 30 µM [1]. Little is currently known about the overall effects of spliceosome inhibition on human cells. Here, we treated HCT116 and a p53 null subline of colon cancer cells with 30 µM IGG for 8 hours. Total RNA was isolated, and Affymetrix oligonucleotide microarray analysis was completed using samples from two biologically independent experiments. A relatively small number of transcripts were differentially expressed in these cell lines. There was considerable overlap in the upregulated but not the downregulated transcripts. PANTHER Reactome analysis of these shared upregulated transcripts identified enriched pathways involving the ATF4 transcription factor important in the integrated stress response [2].

## Specifications Table

**Table:** 

Subject	Genetics: General
Specific subject area	Genome wide changes in gene expression following isoginkgetin treatment
Type of data	Table Chart
How data were acquired	RNA isolation, Affymetrix microarrays, PANTHER gene ontology reactome analysis
Data format	Raw (linked) Analysed
Parameters for data collection	HCT116 and a subline deleted of the p53 tumour suppressor were treated with 30 µM IGG, an equivalent volume of DMSO as a vehicle control, or left untreated for 8 hours.
Description of data collection	Total RNA was isolated using QIAGEN RNeasy mini kits, labelled, hybridized to Affymetrix Human Transcriptome 2.0 oligonucleotide microarrays at the Affymetrix Microarray Facility, Stemcore Laboratory, Ottawa Hospital Research Institute (Ottawa, ON, Canada)
Data source location	Institution: Carleton University City/Town/Region: Ottawa Country: Ontario
Data accessibility	Repository name: Gene Expression Omnibus (GEO) Data identification number: GSE180623 Direct URL to data: https://www.ncbi.nlm.nih.gov/geo/query/acc.cgi?acc=GSE180623 Probe-level files: GSM5466186-GSM466197 Gene-Level files: GSM5466322- GSM5466340
Related research article	van Zyl E, Tolls V, Blackmore A, McKay BC. Isoginkgetin leads to decreased protein synthesis and activates an ATF4-dependent transcriptional response. Biochim Biophys Acta Mol Cell Res. 2021;1868(12):119123.

## Value of the Data

• This microarray data provides information on the transcriptional response of HCT116 and HCT116 p53 null cells following treatment with the pre-mRNA splicing inhibitor, IGG. The data set includes both gene level and individual probe level signal intensities.
• The gene level analysis can be used to gain understanding of the changes in gene expression in the presence and absence of p53 in response to IGG.
• The probe (exon) level analysis can be used to generate additional information on pre-mRNA splicing and alternative splicing.
• The data provided here and linked through the Gene Expression Omnibus (GEO) repository could be used by investigators interested in 1. cellular responses to spliceosome inhibition, 2. the effect of IGG on pre-mRNA splicing and 3. the effects of IGG on non-coding RNAs.

## 1 Data Description

**Fig. 1:**
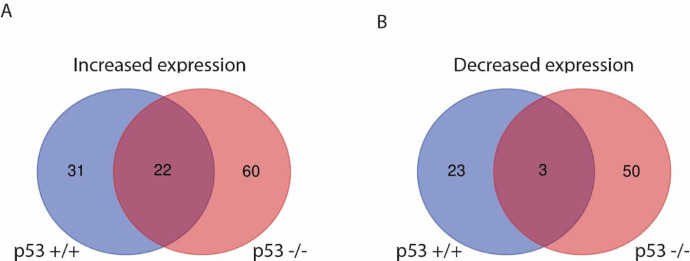
Venn diagrams representing IGG upregulated and downregulated transcripts in HCT116 and its p53 null subline. IGG increased (A) and decreased (B) the expression of mRNAs in HCT116 expressing and deleted of p53 (p53 +/+ and p53 -/-). Images were created from data in Tables 1-4 at https://bioinformatics.psb.ugent.be/webtools/Venn/.

The dataset includes gene level and probe level analysis of the cellular responses to the splicing inhibitor IGG in HCT116 colon cancer cells and an isogenic subline deleted of p53 (HCT116 p53 -/-) [3]. IGG significantly increased the expression of 53 mRNAs and decreased the abundance of 26 in the parental HCT116 cell line (Tables 1 and 2). In the p53 knockout subline, IGG significantly increased the expression of 82 and decreased the expression of 53 mRNAs (Tables 3 and 4). Only 22 of the increased and 3 of the decreased mRNAs were common to both cell lines and therefore they were regulated in a p53-independent manner (Fig. 1A and B). Panther Reactome Analysis of these shared transcripts identified only 3 over-represented pathways among these shared transcripts. Two of the 3 p53-independent responses “Response of EIF2AK1 (HRI) to heme deficiency” and “ATF4 activates genes in response to endoplasmic reticulum stress” involve the ATF4 transcription factor [2].

**Table 1:** List of upregulated transcripts in HCT116 cells following IGG treatment.

Transcript Cluster ID^1^	Gene Symbol^2^	Gene ID^2^	Fold Change^3^
TC15000300.HG.1	CHAC1	79094	6.19
TC16000473.HG.1	MT1F	4494	3.87
TC17001820.HG.1	SLC16A6	9120	3.29
TC10000449.HG.1	DDIT4	54541	3.23
TC08001369.HG.1	SLC10A5	347051	2.46
TC04001570.HG.1	SLC7A11	23657	2.4
TC16000471.HG.1	MT1CP-201	441771	2.31
TC16000476.HG.1	MT1X	4501	2.31
TC14002222.HG.1	IGHD3-22	28497	2.25
TC19000356.HG.1	GDF15	9518	2.19
TC12001625.HG.1	DDIT3	1649	2.08
TC06001089.HG.1	ULBP1	80329	2.05
TC02002445.HG.1	NR4A2	4929	2.01
TC17001723.HG.1	SRSF1	6426	1.95
TC11003457.HG.1	ARL2	402	1.94
TC09002706.HG.1	BAAT	570	1.87
TC16002074.HG.1	MT1M	4499	1.86
TC19000032.HG.1	ATP5D	66043	1.85
TC01000377.HG.1	SESN2	83667	1.82
TC15000461.HG.1	ANXA2	302	1.81
TC07001898.HG.1	CREB3L2	64764	1.78
TC01003796.HG.1	SLC30A1	7779	1.73
TC19000732.HG.1	GFY	100507003	1.71
TC16000057.HG.1	NPW	283869	1.71
TC05002075.HG.1	STC2	8614	1.71
TC19001871.HG.1	TMEM238	388564	1.69
TC0X002235.hg.1	RTL8A	26071	1.66
TC14002240.HG.1	IGHD3-3	28501	1.66
TC16000474.HG.1	MT1H	4496	1.65
TC07001630.HG.1	ASNS	440	1.64
TC16000480.HG.1	HERPUD1	9709	1.63
TC16002035.HG.1	MT1A	4489	1.63
TC22000627.HG.1	XBP1	7494	1.63
TC11002483.HG.1	IFITM2	10581	1.61
TC16002034.HG.1	MT2A	4502	1.61
TC01003194.HG.1	H2AC21	317772	1.6
TC17001617.HG.1	ARL17A	51326	1.6
TC19000156.HG.1	OR7D2	162998	1.6
TC10002938.HG.1	ADIRF	10974	1.59
TC08002271.HG.1	DUSP4	1846	1.57
TC01006390.HG.1	HNRNPU	3192	1.57
TC09000260.HG.1	FAM27E3	100131997	1.56
TC06004075.HG.1	LY6G5B	58496	1.56
TC20000009.HG.1	TRIB3	57761	1.56
TC22000467.HG.1	IGKC	3514	1.55
TC04002952.HG.1	AREG	374	1.54
TC6_QBL_HAP6000133.HG.1	IER3	8870	1.54
TC15002759.HG.1	CKMT1B	1159	1.53
TC06000265.HG.1	HIST1H2BM	8342	1.53
TC05001898.HG.1	PLAC8L1	153770	1.53
TC14002223.HG.1	IGHD2-21	28502	1.52
TC01005437.HG.1	SLC6A9	6536	1.52
TC01001090.HG.1	TXNIP	10628	1.51

1 Transcript Cluster ID assigned from Affymetrix Transcriptome Analysis Console (TAC) 4.0

2 Gene symbols and IDs were obtained from NCBI gene (https://www.ncbi.nlm.nih.gov/gene/)

3 Fold change relative to DMSO vehicle control.

**Table 2:** List of downregulated transcripts in HCT116 cells following IGG treatment.

Transcript Cluster ID^1^	Gene Symbol^2^	NCBI Gene ID^2^	Fold Change^3^
TC17000934.HG.1	CCDC137	339230	-1.51
TC03000672.HG.1	EEFSEC	60678	-1.51
TC12000747.HG.1	ELK3	2004	-1.51
TC17000996.HG.1	METTL16	79066	-1.51
TC22000709.HG.1	TXN2	25828	-1.51
TC17000838.HG.1	CDR2L	30850	-1.52
TC17002052.HG.1	MAP2K4	6416	-1.52
TC03002349.HG.1	PRKCD	5580	-1.52
TC20001762.HG.1	RBM12	10137	-1.52
TC19001036.HG.1	SGTA	6449	-1.52
TC17000162.HG.1	COX10	1352	-1.53
TC20001531.HG.1	BCL2L1	598	-1.54
TC11002866.HG.1	DLAT	1737	-1.54
TC15001257.HG.1	EHD4	30844	-1.56
TC06000796.HG.1	PM20D2	135293	-1.56
TC07003019.HG.1	ABHD11	83451	-1.58
TC6_MANN_HAP4000139.HG.1	C6orf47	57827	-1.58
TC02001139.HG.1	SLC39A10	57181	-1.61
TC0X000321.hg.1	TSR2	90121	-1.62
TC08001507.HG.1	SLC25A32	81034	-1.63
TC16001353.HG.1	CHMP1A	5119	-1.68
TC22001208.HG.1	DGCR2	9993	-1.73
TC16000149.HG.1	PMM2	5373	-1.73
TC12000189.HG.1	EMP1	2012	-1.77
TC05000701.HG.1	EGR1	1958	-1.78
TC12000034.HG.1	TEAD4	7004	-1.92

1 Transcript Cluster ID assigned from Affymetrix Transcriptome Analysis Console (TAC) 4.0

2 Gene symbols and IDs were obtained from NCBI gene https://www.ncbi.nlm.nih.gov/gene/)

3 Fold change relative to DMSO vehicle control.

**Table 3:** List of upregulated transcripts in the HCT116 p53 -/- subline following IGG treatment.

Transcript Cluster ID^1^	Gene Symbol^2^	NCBI Gene ID^3^	Fold Change^4^
TC12001420.HG.1	RNY5	6090	10.21
TC15000300.HG.1	CHAC1	79094	8.44
TC19000356.HG.1	GDF15	9518	5.28
TC17001820.HG.1	SLC16A6	9120	4.77
TC10000449.HG.1	DDIT4	54541	4.66
TC04001570.HG.1	SLC7A11	23657	4.33
TC02002445.HG.1	NR4A2	4929	3.9
TC01003796.HG.1	SLC30A1	7779	3.78
TC08002271.HG.1	DUSP4	1846	3.75
TC05002075.HG.1	STC2	8614	3.18
TC01000377.HG.1	SESN2	83667	2.85
TC16000476.HG.1	MT1X	4501	2.62
TC16000640.HG.1	CMIP	80790	2.6
TC06001089.HG.1	ULBP1	80329	2.59
TC16000473.HG.1	MT1F	4494	2.56
TC01006089.HG.1	SLC30A1	7779	2.53
TC01005688.HG.1	PSMA5	5686	2.42
TC07001898.HG.1	CREB3L2	64764	2.42
TC07001630.HG.1	ASNS	440	2.37
TC08001099.HG.1	DUSP4	1846	2.33
TC15002652.HG.1	ST20	400410	2.23
TC02002818.HG.1	SCG2	7857	2.21
TC16000480.HG.1	HERPUD1	9709	2.18
TC04002953.HG.1	AREG	374	2.03
TC04002952.HG.1	AREG	374	2.02
TC09002484.HG.1	FBXO10	26267	1.98
TC06002799.HG.1	VEGFA	7422	1.96
TC09000358.HG.1	PSAT1	29968	1.95
TC08001701.HG.1	LNCOC1	100288181	1.93
TC11001536.HG.1	CSTF3	1479	1.92
TC06002024.HG.1	TUBE1	51175	1.91
TC02001273.HG.1	PKI55	150967	1.9
TC08002558.HG.1	LNCOC1	100288181	1.88
TC03001929.HG.1	SLC33A1	9197	1.88
TC04001058.HG.1	FGFBP1	9982	1.87
TC22000317.HG.1	ATF4	468	1.87
TC22000627.HG.1	XBP1	7494	1.87
TC05002974.HG.1	RPL37	6167	1.83
TC16000501.HG.1	CCDC113	29070	1.82
TC11003010.HG.1	CARS	27267	1.8
TC0X001317.hg.1	NKAP	79576	1.79
TC11001124.HG.1	GRAMD1B	57476	1.75
TC12001625.HG.1	DDIT3	1649	1.74
TC16001234.HG.1	AARS	234734	1.73
TC10001569.HG.1	AVPI1	60370	1.72
TC16000190.HG.1	C16orf45	89927	1.68
TC03000888.HG.1	PSAT1P4	100287630	1.68
TC01004662.HG.1	SPAG17	200162	1.67
TC11000856.HG.1	PCF11	51585	1.67
TC12003284.HG.1	RHOF	54509	1.65
TC09000508.HG.1	NR4A3	8013	1.64
TC01004603.HG.1	SARS	6301	1.64
TC06002667.HG.1	ZSCAN12P1	221584	1.64
TC09001160.HG.1	FAM27E3	100131997	1.63
TC04000460.HG.1	GPAT3	84803	1.62
TC02002074.HG.1	EIF2AK3	9451	1.61
TC01003555.HG.1	PTP4A1P7	100421681	1.61
TC03001683.HG.1	TMEM39A	55254	1.61
TC07001559.HG.1	SEMA3C	10512	1.6
TC17002686.HG.1	SP2-AS1	100506325	1.6
TC12001976.HG.1	TMEM116	89894	1.6
TC17001617.HG.1	ARL17A	51326	1.6
TC13001319.HG.1	GAS6-AS1	650669	1.59
TC04001496.HG.1	SEC24D	9871	1.59
TC17000396.HG.1	SLFN5	162394	1.58
TC02002067.HG.1	KRCC1	CHBP2	1.57
TC06000056.HG.1	RREB1	6239	1.56
TC02001948.HG.1	GFPT1	2673	1.56
TC17001631.HG.1	SP2-AS1	100506325	1.56
TC20000599.HG.1	GPCPD1	56261	1.56
TC12001282.HG.1	EPS8	DFNB102	1.56
TC09000066.HG.1	LURAP1L	286343	1.55
TC21000284.HG.1	HSPA13	STCH	1.54
TC09000962.HG.1	IFNE	338376	1.54
TC17002458.HG.1	DERL2	51009	1.53
TC16000472.HG.1	MT1B	4490	1.53
TC11001289.HG.1	CARS	27267	1.52
TC16000469.HG.1	MT1JP	4498	1.52
TC11002361.HG.1	HYOU1	10525	1.52
TC09001325.HG.1	NFIL3	4783	1.52
TC22000477.HG.1	MIR3198-1	100423025	1.52
TC15002699.HG.1	AP3S2	10239	1.52
TC01002060.HG.1	SREBF2-AS1	112637020	1.51
TC04002517.HG.1	UGDH	7358	1.51
TC20001122.HG.1	CSRP2BP	100303755	1.51
TC01003623.HG.1	EDEM3	80267	1.51
TC09001589.HG.1	HSPA5	3309	1.51
TC16000474.HG.1	MT1H	4496	1.5

1 Transcript Cluster ID assigned from Affymetrix Transcriptome Analysis Console (TAC) 4.0

2 Official gene symbol from NCBI gene webpage

3 Gene ID identifiers from NCBI gene webpage

4 Relative fold changes compared to DMSO vehicle control, analyzed using Affymetrix Transcriptome Analysis Console (TAC) 4.0

**Table 4:** List of downregulated transcripts in the HCT116 p53 -/- subline following IGG treatment.

Transcript Cluster ID^1^	Gene Symbol^2^	NCBI Gene ID^3^	Fold Change^4^
TC10000577.HG.1	LINC00857	6659	-1.51
TC16001601.HG.1	CMTM3	84056	-1.51
TC07002499.HG.1	SERPINE1	89795	-1.51
TC14001209.HG.1	SGPP1	6558	-1.51
TC11003273.HG.1	PCF11-AS1	7025	-1.52
TC17000692.HG.1	STXBP4	730755	-1.52
TC12001637.HG.1	CTDSP2	84159	-1.52
TC14000353.HG.1	DACT1	51339	-1.54
TC02000192.HG.1	CLIP4	79745	-1.54
TC13000431.HG.1	GAS6-AS2	100506394	-1.55
TC17002807.HG.1	TIMP2	7077	-1.55
TC04000883.HG.1	MIR1305	100302270	-1.55
TC18000205.HG.1	MALT1	10892	-1.56
TC0X001397.hg.1	MOSPD1	56180	-1.57
TC05002969.HG.1	DAB2	1601	-1.58
TC07002368.HG.1	ZNF107	51427	-1.59
TC09002524.HG.1	ANKRD20A3	441425	-1.6
TC03002235.HG.1	NR2C2	7182	-1.61
TC09000999.HG.1	DDX58	23586	-1.61
TC17001532.HG.1	PTRF	284119	-1.61
TC01000353.HG.1	SFN	2810	-1.61
TC03002676.HG.1	ACTL6A	86	-1.62
TC17002855.HG.1	AXIN2	8313	-1.63
TC05003395.HG.1	TRIM52	84851	-1.63
TC03003038.HG.1	ZBTB20	26137	-1.63
TC10000377.HG.1	ARID5B	84159	-1.63
TC04001830.HG.1	TRIML2	205860	-1.64
TC03002740.HG.1	FAM43A	131583	-1.64
TC03001512.HG.1	ID2B	84099	-1.64
TC04002781.HG.1	SH3D19	152503	-1.65
TC04001615.HG.1	ZNF827	152485	-1.67
TC07001980.HG.1	CTAGE4	100128553	-1.67
TC05000795.HG.1	SH3RF2	153769	-1.67
TC17001803.HG.1	SMURF2	64750	-1.69
TC04002771.HG.1	ZNF827	152485	-1.73
TC17001722.HG.1	VEZF1	7716	-1.74
TC10002813.HG.1	CALHM2	51063	-1.74
TC14002241.HG.1	IGHD2-2	28505	-1.74
TC19002012.HG.1	UCA1	652995	-1.74
TC12002734.HG.1	SLC2A3	6515	-1.74
TC03001959.HG.1	SPTSSB	165679	-1.75
TC11000715.HG.1	MYEOV	26579	-1.85
TC12000189.HG.1	EMP1	2012	-1.86
TC05000218.HG.1	ITGA2	3673	-1.88
TC07001605.HG.1	SAMD9	54809	-1.9
TC02001139.HG.1	SLC39A10	57181	-1.92
TC15002251.HG.1	SMAD3	4088	-1.94
TC12000747.HG.1	ELK3	2004	-1.95
TC05002612.HG.1	SLC12A2	6558	-1.99
TC12000656.HG.1	NAV3	89795	-2.00
TC13001403.HG.1	KATNAL1	84056	-2.02
TC06000135.HG.1	SOX4	6659	-2.11
TC10002092.HG.1	ARID5B	84159	-2.25
TC17001485.HG.1	KRTAP2-3	730755	-2.48
TC05002512.HG.1	NR2F1	7025	-2.52

1 Transcript Cluster ID assigned from Affymetrix Transcriptome Analysis Console (TAC) 4.0

2 Official gene symbol from NCBI gene webpage

3 Gene ID identifiers from NCBI gene webpage

4 Relative fold changes compared to DMSO vehicle control, analyzed using Affymetrix Transcriptome Analysis Console (TAC) 4.0

**Table 5:** Summary of Panther Reactome analysis of transcripts induced by IGG in both cell lines.

Reactome pathway^1^	Enrichment^2^	P value^3^
Metallothieoneins bind metals	>100	7.88E-04
>Response to metal ions	>100	1.47E-03
Response to EIF2AK1 (HRI) to heme deficiency	>100	1.76E-03
ATF4 activates genes in response to ER stress	>100	8.69E-03
>PERK regulates gene expression	87.76	1.40E-02
>Unfolded protein response	41.15	6.00E-03

1 Panther Reactome Pathways that were enriched. ‘>’ indicates that the Reactome Pathway above is nested.

2 Fold enrichment above expected.

3 The probability of observing this enrichment in a random list of genes of this size determined by Fisher exact test with Bonferroni correction for multiple testing.

## 2 Experimental Design, Materials and Methods

### 2.1 Cell culture and drug treatment

HCT116 and HCT116 p53-/- colon cancer cells were seeded in 6cm dishes at 250,000 cells/dish 24 hours prior to drug treatment. Cells were treated with 30 µM IGG, an equivalent volume of DMSO as a vehicle control, or left untreated for 8 hours. Two biologically independent experiments were performed.

### 2.2 RNA isolation and microarrays

After 8 hours of treatment, media was removed, and cells were washed with PBS. Total RNA was isolated using the Qiagen RNAeasy Mini RNA isolation kit according to manufacturer's instructions. RNA purity and concentration was determined using the DeNovix DS-11 spectrophotometer. RNA was sent for Agilent Bioanalyzer quality assessment and RNA was then processed for analysis of the Human Transcriptome 2.0 Array at the Stemcore facility at the Ottawa Hospital research institute (OHRI), Ottawa, ON Canada.

### 2.3 Data analysis

Analysis was performed at the probe- and gene-level using the Transcriptome Analysis Console (TAC) 4.0 Software from Affymetrix. Microarray data was analysed using the Affymetrix Transcriptome Analysis Console (TAC) 4.0 software with default settings. A gene was considered expressed in a particular condition if it was detected in 50% of more of the samples and the sample had a DABG p-value of less than 0.05. A one-way between-subject unpaired ANOVA was used to determine statistical significance and was subject to false discovery rate (FDR) multi-test correction (Benjamini-Hochberg Step-Up FDR) for both analyses. A threshold of a 1.5-fold change was also applied and unknown transcripts were removed to identify upregulated (Tables 1 and 3) and downregulated (Tables 2 and 4) transcripts. Panther reactome analysis of RNAs induced in both cell lines (Fig. 1A) was performed online (http://geneontology.org/).

## CRediT Author Statement

**Erin van Zyl:** Formal analysis, Investigation, Writing – original draft, Writing – review & editing; **Victoria Tolls:** Methodology, Investigation, Formal analysis; **Bruce McKay:** Conceptualization, Methodology, Formal analysis, Resources, Writing – review & editing, Supervision, Funding acquisition.

## Declaration of Competing Interest

The authors have no competing interests to declare

## Data Availability

Microarray analysis of isoginkgetin-treated HCT116 and HCT116p53-/- cells (Original data) (NCBI GEO).
